# Cache Domain Containing 1 Is a Novel Marker of Non-Alcoholic Steatohepatitis-Associated Hepatocarcinogenesis

**DOI:** 10.3390/cancers13061216

**Published:** 2021-03-10

**Authors:** Anna Kakehashi, Arpamas Chariyakornkul, Shugo Suzuki, Napaporn Khuanphram, Kumiko Tatsumi, Shotaro Yamano, Masaki Fujioka, Min Gi, Rawiwan Wongpoomchai, Hideki Wanibuchi

**Affiliations:** 1 Department of Molecular Pathology, Graduate School of Medicine, Osaka City University, Abeno-ku 1-4-3 Asahi-machi, Osaka 545-8585, Japan; suzuki.shugo@med.osaka-cu.ac.jp (S.S.); ktatsumik@gmail.com (K.T.); syamano@ncc.go.jp (S.Y.); m2066048@med.osaka-cu.ac.jp (M.F.); mwei@med.osaka-cu.ac.jp (M.G.); wani@med.osaka-cu.ac.jp (H.W.); 2 Department of Biochemistry, Faculty of Medicine, Chiang Mai University, 110 Inthawarorot Rd., Sri Phum, Muang, Chiang Mai 50200, Thailand; arpamas_ch@cmu.ac.th (A.C.); napaporn_khuan@cmu.ac.th (N.K.); rawiwan.wong@cmu.ac.th (R.W.)

**Keywords:** CACHD1, hepatocarcinogenesis, NASH, HCC, STAM mice

## Abstract

**Simple Summary:**

The aim of the present study was to discover novel early molecular biomarkers of liver neoplasms which arise in non-alcoholic steatohepatitis (NASH) Stelic Animal Model (STAM) mice. Significant increase of lipid deposits, hepatocyte ballooning, fibrosis, and incidences and multiplicities of hepatocellular adenomas and carcinomas were detected in the livers of 18-week-old STAM mice. From the results of proteome analysis of STAM mice hepatocellular carcinomas, significant elevation of a novel protein, cache domain-containing 1 (CACHD1) was found. Furthermore, we observed CACHD1-positive foci in STAM mice livers, which number, area, and cell proliferation index within the foci were significantly elevated. Results of immunohistochemical and in vitro functional analysis indicated that CACHD1 may become a useful early biomarker and potential molecular target in NASH-associated hepatocarcinogenesis, which is involved in control of cell proliferation, autophagy and apoptosis.

**Abstract:**

In the present study, potential molecular biomarkers of NASH hepatocarcinogenesis were investigated using the STAM mice NASH model, characterized by impaired insulin secretion and development of insulin resistance. In this model, 2-days-old C57BL/6N mice were subjected to a single subcutaneous (s.c.) injection of 200 μg streptozotocin (STZ) to induce diabetes mellitus (DM). Four weeks later, mice were administered high-fat diet (HFD) HFD-60 for 14 weeks (STAM group), or fed control diet (STZ group). Eighteen-week-old mice were euthanized to allow macroscopic, microscopic, histopathological, immunohistochemical and proteome analyses. The administration of HFD to STZ-treated mice induced significant fat accumulation and fibrosis development in the liver, which progressed to NASH, and rise of hepatocellular adenomas (HCAs) and carcinomas (HCCs). In 18-week-old animals, a significant increase in the incidence and multiplicity of HCAs and HCCs was found. On the basis of results of proteome analysis of STAM mice HCCs, a novel highly elevated protein in HCCs, cache domain-containing 1 (CACHD1), was chosen as a potential NASH-HCC biomarker candidate. Immunohistochemical assessment demonstrated that STAM mice liver basophilic, eosinophilic and mixed-type altered foci, HCAs and HCCs were strongly positive for CACHD1. The number and area of CACHD1-positive foci, and cell proliferation index in the area of foci in mice of the STAM group were significantly increased compared to that of STZ group. In vitro siRNA knockdown of CACHD1 in human Huh7 and HepG2 liver cancer cell lines resulted in significant inhibition of cell survival and proliferation. Analysis of the proteome of knockdown cells indicated that apoptosis and autophagy processes could be activated. From these results, CACHD1 is an early NASH-associated biomarker of liver preneoplastic and neoplastic lesions, and a potential target protein in DM/NASH-associated hepatocarcinogenesis.

## 1. Introduction

Nowadays, multisystem diseases such as nonalcoholic fatty liver disease (NAFLD) and steatohepatitis (NASH) have become a common cause of morbidity and mortality from cirrhosis, liver failure and hepatocellular carcinoma (HCC) worldwide. In humans, NASH is known to be directly associated with obesity and several intestinal and metabolic diseases, including diabetes mellitus (DM), and does have histological features in the liver, such as fat deposition, inflammation and fibrosis. Nevertheless, recent evidence signifies that HCC development due to NASH is observed in both obese and non-obese patients dependent on various genetic and environmental factors. The accumulation of lipids in the liver, alterations to leptin, adiponectin and adipocytokines derived from adipose tissue, the development of oxidative and endoplasmic reticulum (ER) stresses, mitochondrial dysfunction, gut and bile duct-associated inflammation, presence of a variant of *transmembrane 6 superfamily member 2* gene (*TM6SF2*) and the influence of some drugs are considered as common chronic conditions predisposing to NASH onset in the liver [1,2,3]. Activation of β-catenin, SMAD3-transforming growth factor-β (SMAD3-TGF-β), nuclear factor (erythroid-derived 2)-like 2 (Nrf2), sterol regulatory element-binding protein and liver X receptor α (SREBP-LXRα) and nuclear receptor-interacting protein 1 (NRIP1), along with the inhibition of peroxisome proliferator-activated receptors (PPARs) and tumor suppressor p53 in human NASH biopsies and HCCs has been reported [4]. However, the differential mechanisms of NASH development and its progression to HCC remain to be elucidated.

In previous studies, mechanisms of progression from NASH to HCC were suggested to differ depending on the risk factors; therefore, several animal NASH models have been recently developed. Long-term feeding with HFD was shown to be associated with obesity and hepatic steatosis, insulin resistance, fibrosis and development of hepatic tumors; however, the severity of liver damage induced by HFD is low and varies with the mouse strain [5,6]. Other NASH models included methionine and choline-deficient diet (MCDD), choline-deficient high-fat diet (CHFD) [7,8] and choline-deficient, L-amino acid-defined, high-fat diet (CDAHFD) models [9], in which the metabolic syndrome features are lacking. In diet models, methionine and choline deficiency leads to extensive hepatic lipid accumulation, fibrosis and steatohepatitis; both of them are important for production of very low-density lipoprotein (VLDL) [7]. In our recent studies, we performed CDAHFD NASH model, and Tsumura, Suzuki, Obese Diabetic (TSOD) mouse metabolic syndrome model with type 2 diabetes (T2DM), established by selective breeding of ddY mice [10,11]. In TSOD mouse NASH model, the specific activation of mTOR pathway in HCC was found, whereas no activation was obvious in CDAHFD-treated C57Bl/6J mice [12], highlighting differences in mechanisms of NASH hepatocarcinogenesis in metabolic syndrome and CDAHFD models.

Among animal models, to study the molecular mechanisms of hyperglycemia/hyperlipidemia-associated NASH hepatocarcinogenesis, Stelic Animal Model (STAM) has attracted attention. STAM mice do not become obese, but exhibit the progressive loss of insulin production and development of diabetes mellitus (DM). In this NASH model, HCC induction occurs at a very high rate and it is particularly useful to study the progression of hepatic fibrosis to cirrhosis and HCC. To induce DM and NASH, neonatal 2-days-old C57BL/6N mice are administered subcutaneous (s.c.) injection of antibiotic streptozotocin (STZ), which is particularly toxic to pancreatic β cells, and administered HFD from 4 weeks of age. Histopathological characteristics of NASH, such as microvesicular fat and hepatocellular ballooning are observed from 4 weeks of age [13]. Insulin resistance was observed in STAM mice, which is developed due to the induced DM [13]. As the administration of STZ at high dose was reported to exert hyperglycemia-independent direct hepatotoxic effects [14], to minimize the STZ effect on the liver, the breeding company has employed careful dose titration. The increase in oxidative stress with hyperglycemia has been suggested to trigger hepatic lesions in STAM mice, while insulin resistance promotes lesion formation with hepatic lipid accumulation [15]. Thus, rapid development of hyperglycemia and inflammation was accompanied by significant hepatic pathological changes associated with NASH, such as hepatosteatosis, hypertrophic hepatocytes and fibrosis at 10 weeks of age, which rapidly progress to cirrhosis at 12 weeks, and eventually to HCC at 18 weeks with the incidence of about 100% in males [13]. However, the concrete hepatocarcinogenesis mechanisms and preneoplastic lesions markers in STAM mice NASH model remain unknown.

In the present study, we were particularly interested to find novel early molecular biomarkers of liver neoplasms, which arise as a consequence of NASH in STAM mouse model. Proteome analysis in 18-week-old STAM mice HCCs was performed for identification of specific biomarkers. After that, immunohistochemical verification in vivo using STAM mice NASH model and the molecular functional analysis in vitro were conducted.

## 2. Results

### 2.1. STAM Mice NASH Model

#### 2.1.1. General Conditions

**Figure 1:**
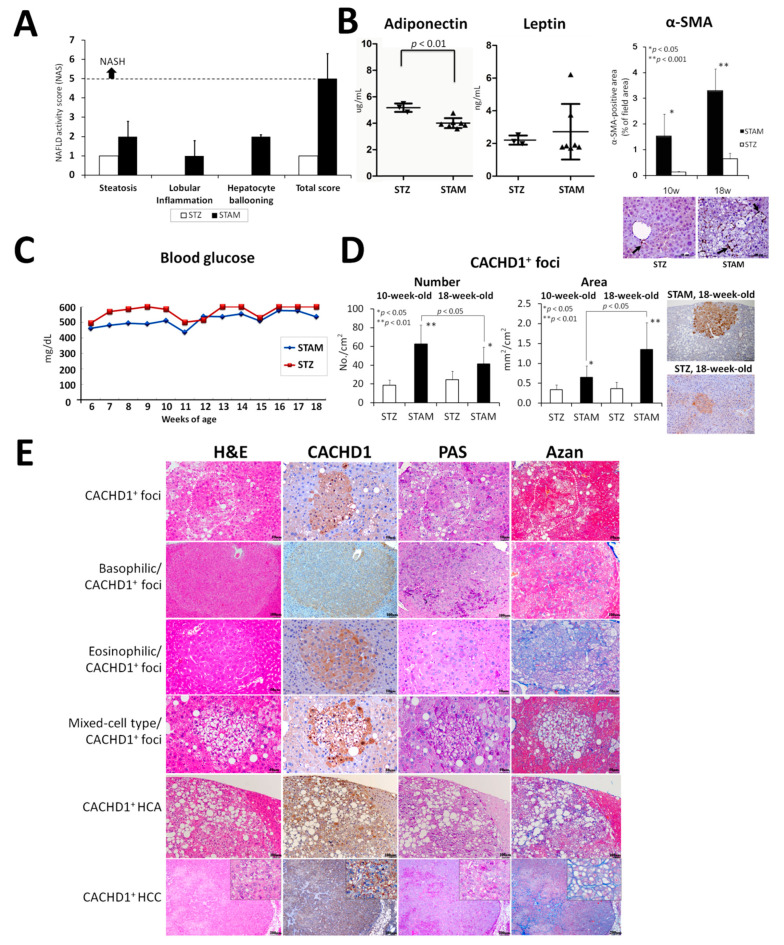
(**A**) Steatosis, lobular inflammation, hepatocyte ballooning and total non-alcoholic fatty liver disease (NAFLD) activity scores in 18-week-old Stellic Animal Model (STAM) and streptozotocin (STZ) control mice. (**B**) Serum blood adiponectin, leptin and α-SMA levels in 18-week-old mice, (**C**) Blood glucose levels, (**D**) Numbers and areas of CACHD1^+^ foci developed in 10 and 18-weeks old mice. (**E**) Representative pictures of H&E, PAS, AZAN staining, and CACHD1 immunohistochemistry in the livers of STAM mice. CACHD1^+^ preneoplastic lesions included basophilic, eosinophilic, mixed-cell type, and those which were undetectable histopathologically. Note the elevation of PAS-positive (glycogenation) areas in all lesions, Azan-positive (fibrosis) areas in AF (mainly eosinophilic and mixed-cell type foci) and liver tumors, and strongly positive for CACHD1 nuclei and cytoplasm of the ballooned cells in CACHD1^+^ alone and mixed-cell type foci. *, *p* < 0.05, **, *p* < 0.01. Scale Bar: 100 μm (**B**,**D**); 50 μm, 100 μm and 200 μm in foci, HCA and HCC images, respectively, in (**E**).

No mice died during the experiment, and no significant differences were observed in the body weights. No significant differences in relative liver weights were detected in 10- and 18-week-old STAM mice administered HFD compared with STZ-treated control mice. STAM mice were non-obese and developed NASH phenotype (Figure 1A). Low levels of steatosis (microvesicular fat accumulation), hepatocyte hypertrophy and vacuolation were observed in the livers of STZ control mice, due to persistent DM. In our study, such abnormalities as liver lobular inflammation and hepatocyte ballooning were found only in the livers of STAM mice administered HFD but not in STZ control age-matched littermates (Figure 1A). The total NAFLD activity score was highly elevated in the livers of 18-week-old STAM mice compared with STZ control animals (Figure 1A). Furthermore, adiponectin level was significantly lower in the blood of STAM mice compared with the STZ control group; however, leptin levels did not differ in both groups (Figure 1B). In addition, a significantly higher level of smooth muscle actin (α-SMA) in the livers of STAM mice at 18 weeks of age was detected using IHC, signifying an increase in fibrosis (Figure 1B). Fasting blood glucose levels were similarly elevated in both STAM and STZ control mice being indicative of DM development (Figure 1C).

#### 2.1.2. Histopathological Analysis

Results of histopathological examination in STAM and STZ control mice are presented in Table 1 and Figure 1E. No tumors were detected in the liver of STZ control mice; however, few basophilic, eosinophilic and mixed-cell type (vacuolated/clear-cell) altered foci (AF) were obvious. The reasons for AF development in the STZ-treated mice livers could be the rise in hyperglycemia, steatosis and induction of low levels of oxidative stress as previously reported by examination of hexanoyl-lysine (HEL) oxidative stress markers [16]. In contrast, numerous hepatocellular adenomas (HCAs) and HCCs containing many lipid droplets and glycogen granules in tumor cells were found in 18-week-old HFD-administered STAM mice livers. HCAs were round, with few mitotic figures, but HCCs were actively proliferating, their structure was heteromorphic, featuring both large and small tumor cells, furthermore, oval cells and infiltrating inflammatory cells were observed. Incidence and multiplicity of HCC in STAM mice at 18 weeks of age were significantly higher than in the STZ control group (Table 1).

#### 2.1.3. Proteome Analysis of STAM Mice HCCs

Results of proteome analysis of differentially expressed proteins with more than a 2-fold increase of expression in 18-week-old STAM mice HCCs compared with STZ control mouse liver are presented in Table 2.

We searched for the most highly overexpressed proteins, which could become potential biomarkers in STAM mice DM/NASH hepatocarcinogenesis. As a result, a novel 3.6-fold elevated protein, cache domain-containing 1 (CACHD1), which is known to be involved in calcium ion transport affecting neurotransmission in the central nervous system, attracted our attention [17,18,19]. In our previous study, no CACHD1 protein expression was detected in non-treated control C57BL/6N mice [4]. In addition, the elevation of CACHD1 detected in STAM mice HCCs was coordinated with the significant increase in intermediate filament proteins CK8 and CK18, mitochondrial proteins and transcriptional regulators prohibitins 1 and 2, enzymes glutathione S-transferase Mu 1 (GSTM1), peroxisomal bifunctional enzyme (EHHADH) and mitochondrial ornithine aminotransferase (OAT), which are involved in glutathione metabolism, fatty acid β-oxidation in peroxisomes and catabolism of arginine, respectively.

#### 2.1.4. Immunohistochemical Assessment of CACHD1 in Mice Livers

Immunohistochemical assessment of CACHD1 in STAM and STZ control mice livers at 10 and 18 weeks of age demonstrated significant elevation of number and area of CACHD1-positive (CACHD1^+^) foci in the livers of STAM mice compared to the STZ control group (Figure 1D and E). CACHD1 expression was low in the livers of STZ control mice, and was bound to the liver areas with steatosis. Furthermore, high levels of CACHD1 were found in the STAM mice preneoplastic lesions and tumors. Comparison of hematoxylin and eosine (H&E) and CACHD1-stained serial slides demonstrated that most of the developed altered foci (AF) (basophilic (BF), eosinophilic (EF) and mixed-cell (vacuolated/clear-cell) (MF)) type in 18-week-old STAM and STZ mice were positive for CACHD1 (Table 3 and Figure 1E). Very few of them were CACHD1-negative. Interestingly, in the livers of STAM mice, we detected strongly stained CACHD1^+^ foci, which were impossible to identify histopathologically by H&E staining (Figure 1D and Table 2). Those foci were small and numerous in 10-week-old STAM mice, but their size increased and the number decreased in 18-week-old STAM mice, likely due to the development of liver tumors from some of them. CACHD1 was strongly overexpressed in non-BF/EF/MF, mixed-cell type and eosinophilic AF, but its staining was less pronounced in basophilic foci. Furthermore, in non-BF/EF/MF type and mixed-cell type foci, strong CACHD1 overexpression was found in both the nuclear and the cytoplasm (mainly ballooned and clear cells), while in basophilic and eosinophilic foci, CACHD1 was observed only in the cytoplasm (Figure 1E). All HCAs and HCCs developed in 18-week-old STAM mice were positive for CACHD1. In tumors, CACHD1 was localized in the cell nuclear, cytoplasm or both of them.

**Figure 2:**
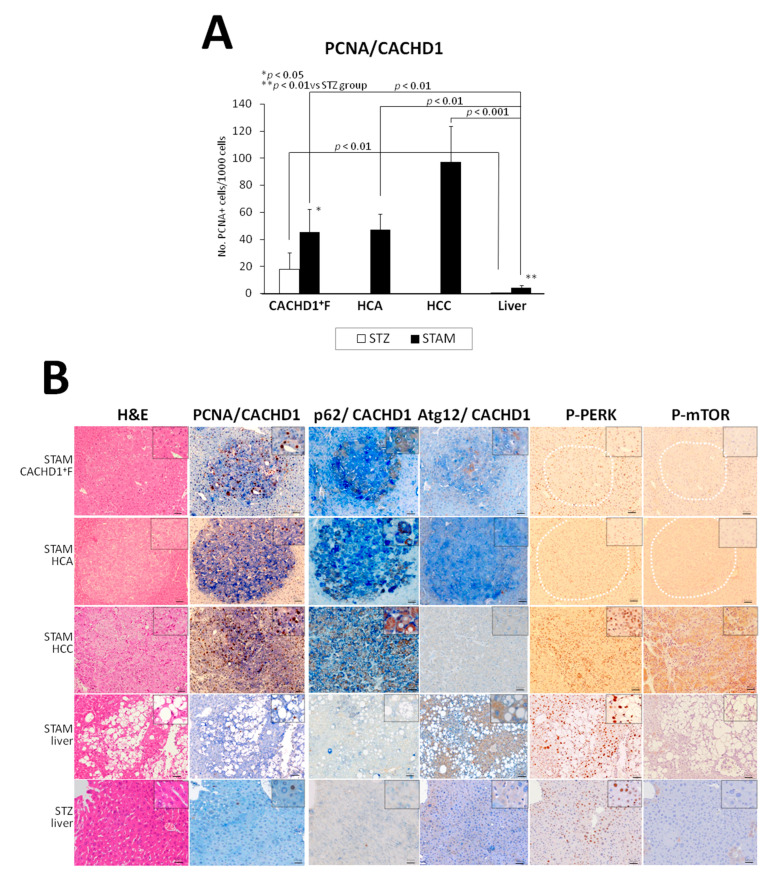
Alterations of cell proliferation (PCNA) (**A**) in the area of CACHD1^+^ foci, HCAs, HCCs, and livers of 18-week-old STAM and STZ control mice. (**B**) Representative pictures of double IHC for PCNA/CACHD1, p62/CACHD1, Atg12/CACHD1, and single IHC for P-PERK and P-mTOR in STAM and STZ control mice. Note the elevation of PCNA and p62 in CACHD1^+^ foci, HCAs and HCCs, strong Atg12 and P-PERK positivity in the surrounding liver of STAM mice and lower expression in CACHD1^+^ foci and tumors, and P-mTOR-positive staining of STAM mice HCC. Scale Bar: 50 μm.

Representative pictures of H&E staining and results of double and single IHC investigation of CACHD1 and cell proliferation, autophagy markers in mice liver AFs, HCAs and HCCs are presented in Figure 2. Investigation of the expression of cell proliferation marker, proliferating cell nuclear antigen (PCNA), and CACHD1 in the livers of mice by double IHC, revealed a significant elevation of PCNA-positive cell number in CACHD1^+^ foci, HCAs and HCCs compared with surrounding liver tissue in 18-week-old STAM and STZ control mice (Figure 2A,B).

Elevation of ubiquitin-binding protein p62 (p62) protein, a classical receptor of autophagy, whose expression is decreased due to its inhibition [20], was observed in CACHD1^+^ foci, HCAs and HCCs. In contrast, autophagy marker *ubiquitin-like proteins autophagy-related* genes 12 (*Atg12*) and *7 Atg7* [21], which form a complex, and phosphorylated form of protein kinase-like endoplasmic reticulum kinase (P-PERK), a marker of NASH-associated endoplasmic reticulum (ER) stress [22], were both highly overexpressed in the surrounding liver of STAM mice, but their expression was reduced in CACHD1^+^ foci, HCAs and HCCs of STAM mice (Figure 2B). In addition, phosphorylated form of mammalian target of rapamycin (mTOR) (P-mTOR), involved in cell growth, survival, metabolism and angiogenesis, was significantly overexpressed in the areas of CACHD1^+^ HCCs, but negative in surrounding livers, AF and HCAs, indicating that mTOR activation occurs lately in NASH hepatocarcinogenesis in STAM mice (Figure 2B).

### 2.2. In Vitro Functional Analysis

#### 2.2.1. Effects Induced by CACHD1siRNAs Knockdown in Huh7 and HepG2 Cells

**Figure 3:**
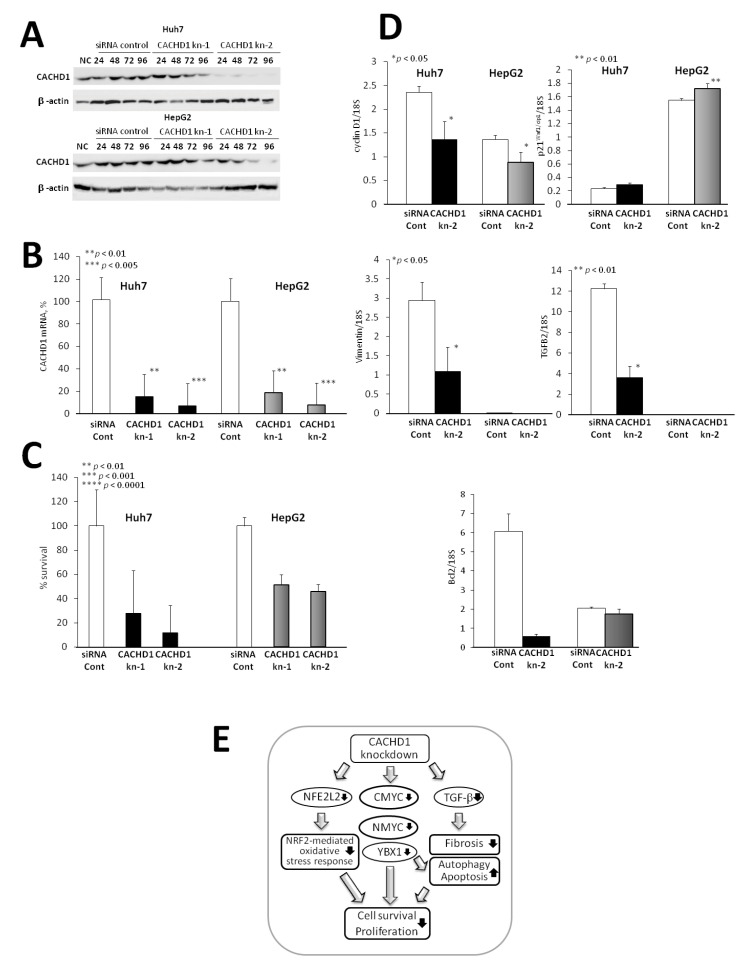
Effects of CACHD1 siRNA knockdown in Huh7 and HepG2 human hepatoma cell lines. Western blot (**A**) and RT-PCR analysis (**B**) of CACHD1 expression, (**C**) cell viability detected by WST8 assay, (**D**) cyclin D1 and p21^Waf1/Cip1^ mRNA expression in CACHD1kn-2 cell lines, and (**E**) results of upstream regulator and pathway analysis by IPA in CACHD1kn Huh7/HepG2 cells. Note the significant suppression of cell viability, decrease of cyclin D1 and elevation of p21^Waf1/cip1^ mRNA expression in CACHD1kn cell lines. Uncropped Western Blot of subfigure (**A**) is available in Figure S1.

Because of limitations including availability and donor variability, the Huh7 and HepG2 are commonly used for investigating lipid metabolism in NAFLD and NASH. In this study, we therefore examined protein levels of CACHD1 in Huh7 and HepG2 cells, and confirmed CACHD1 positivity in both of them. A significant reduction of CACHD1 protein level in both Huh7and HepG2 cells with the transfection of si-CACHD1kn-1 and si-CACHD1kn-2 was confirmed by Western blot analysis (Figure 3A and Figure S1). Furthermore, analysis of CACHD1 mRNA expression in knockdown cells by real-time PCR revealed significantly suppressed levels, as compared with cells transfected with control siRNA (Figure 3B). si-CACHD1kn-2 have shown better knockdown efficiency.

Knockdown of CACHD1 by si-CACHD1kn-1 and si-CACHD1kn-2 exerted an inhibitory effect on cell survival compared with siRNA control, as detected by WST-8 assay (Figure 3C).

Furthermore, the suppression of cyclin D1 (CD1) mRNA expression was demonstrated by RT-PCR analysis in both CACHD1kn-2 Huh7 and HepG2 liver cancer cell lines (Figure 3D). A trend for increase and a significant elevation of p21^Waf1/Cip^ expression was detected in CACHD1kn-2 Huh7 and HepG2 cells, respectively (Figure 3D). Moreover, significant inhibition and a strong trend for the decrease of TGFB2 and vimentin were found in CACHD1kn Huh7 and HepG2 cells, respectively (Figure 3D).

#### 2.2.2. Proteome and Ingenuity Pathway Analyses of CACHD1 Knockdown Huh7 and HepG2 Cell Lines

Effect of CACHD1 knockdown on the proteome of Huh7 and HepG2 human liver cancer cell lines were investigated by QSTAR LC-Ms/Ms, and similar alterations in protein spectra were detected in Huh7 and HepG2 cells using the si-CACHD1kn-2. Proteins with significantly suppressed expression identified in non-label and iTRAQ label proteome analyses in Huh7 cell line are listed in Table 4. CACHD1 knockdown strongly inhibited the expression of proteins involved in protein folding, unfolded protein response (calreticulin (CALR), calumenin (CALU) and heat shock 70 kDa proteins 2, 9 and 5) and cytoskeleton organization including components of intermediate filaments (CK8, CK18, CK19) and actin cytoskeleton (myristoylated alanine-rich protein kinase C substrate (MARCKS), MARCKS-like 1 (MARCKSL1), cofilin 1(CFL1) and profilin 1 (PFN1) (Table 4). In CACHD1kn cells, the expression of stress response-associated proteins, such as superoxide dismutase 2 (SOD2), epoxide hydrolase 1 (EPHX1), nucleophosmin (NPM1), Y box binding protein 1 (YBX1), serological liver disease and HCC markers, aspartate aminotransferase 2 (GOT2) and alpha-fetoprotein (AFP), expression was decreased, while the expression of autophagy inducers, nucleolin (NCL) and Lemur tyrosine kinase 3 (LMTK3), was elevated.

Results of up-stream regulator and pathway analyses detected by Ingenuity Pathway analysis (IPA) are presented in Figure 3E. Canonical pathway analysis by IPA demonstrated that in CACHD1kn Huh7 and HepG2 cell lines Nrf-2-mediated oxidative protein response, unfolded protein response, intermediate and actin cytoskeleton organization were inhibited. Upstream regulator analyses by IPA based on alterations of differentially expressed proteins in STAM mice HCCs have predicted that common transcriptional factors suppressed by CACHD1 knockdown, which drive the expression changes, are proto-oncogenes *c-myc* (*CMYC*), *n-myc* (*NMYC*), nuclear factor (erythroid-derived 2)-like 2 (NFE2L2 (Nrf2)), TGF-β and Y box binding protein 1 (YBX1) (Figure 3E).

## 3. Discussion

In the present STAM mouse NASH model, overexpression of the novel protein, CACHD1 was first recognized in HCCs of 18-week-old mice with the help of proteome analysis, and thereafter CACHD1^+^ foci and tumor formation was immunohistochemically confirmed in the livers of 10- and 18-week-old STAM mice. Although no CACHD1^+^ foci in the mouse liver was earlier found in NASH animal models, a marked increase in their number and area was here evident, demonstrating that this could be an early event in STAM mice NASH hepatocarcinogenesis. Our results indicated a conspicuous CACHD1 increase of nuclear and cytoplasmic localization in histologically detectable and undetectable foci of cellular alteration, HCAs and HCCs of STAM mice. Importantly, cellular proliferation was significantly elevated, but autophagy was likely to be suppressed in both CACHD1^+^ foci and liver tumors of STAM mice.

At present, there are few reports investigating the cellular function of CACHD1protein. CACHD1 is the putative calcium (Ca^2+^) channel and chemotaxis receptor, which has structural similarities with the members of the α_2_δ voltage-gated Ca^2+^ channel auxiliary subunit family [18]. Cottrell et al. demonstrated that CACHD1 protein and mRNA are elevated in the male mammalian central nervous system (CNS), mainly in the thalamus, hippocampus, cerebellum, and hippocampal neurons, where it induces an increase in neuronal firing [18]. Furthermore, CACHD1 was shown to increase the presence and form complexes with Ca^2+^ channel CaV3.1 at the cell surface, and increase channel open probability. It has also been suggested to co-immunoprecipitate with Ca^2+^ channel CaV2.2 and to influence its trafficking and function [17]. Moreover, CACHD1 has been recently identified as a substrate of γ-secretase in CNS, which is well-known for its role in Notch signaling and in Alzheimer’s disease, where it catalyzes the formation of the pathogenic amyloid beta (Abeta) peptide [23,24], which modulates voltage-gated Ca^2+^ channel activity [17,18,19]. CACHD1 was further reported to be the novel in vivo substrate for the protease beta-site APP cleaving enzyme 1 (BACE1), suggesting that its cleavage contributes to the numerous functions of BACE1 in the nervous system [23]. CACHD1 expression was reported to be regulated by cytochrome P450 isoenzymes CYP1B1, CYP1A1 and CYP1A2 [25]. Mutations of *CACHD1* gene were detected in human colorectal adenocarcinoma, malignant melanoma, astrocytoma and oligodendroglioma [26,27,28]. Most recently, CACHD1 was found up-regulated in soft tissue sarcoma, namely, malignant peripheral nerve sheath tumor (MPNST) cells (BL1391) [29]. In this study, we present the first evidence of CACHD1 overexpression in NASH-associated hepatomas and liver preneoplastic lesions in mice.

Induction of diabetes is thought to be essential for the activation of carcinogenic properties of liver cells; however, the concrete mechanisms are still unknown due to the difficulties to discover alterations in liver histopathological changes in patients with diabetes [30]. Continuous administration of HFD to STZ-treated mice has been shown to cause increased lobular inflammation with infiltrated macrophages, which progressed to severe “chicken-wired” fibrosis at 14 weeks, and later to exhibit higher levels of alpha-fetoprotein-positive HCC formation at 18 weeks [31]. The increase of hyperglycemia with oxidative stress was further suggested to trigger hepatic lesions in STAM mice, whereas insulin resistance promoted lesion formation with hepatic lipid accumulation [16]. In addition, the difference in the mRNA expression of serine palmitoyltransferase 3 (Sptlc3), an enzyme involved in the pathway of sphingolipid metabolism in STAM mice livers was found, and this was potentially associated with NASH progression over time [31]. It has been reported that male C57Bl/6J mice treated with a low dose of STZ alone showed diabetes with the absence of NASH-based fibrosis, and, therefore, never developed HCC [13]. In the present study, we, however, detected a slight increase of steatosis, α-SMA and development of few AF in STZ control mice livers.

In this study, coordinated overexpression of CACHD1 and intermediate filament members CK8, CK18, actin-related proteins such as SEPT9, mitochondrial proteins including prohibitins and YME1L1, and proteins involved in protein folding and unfolded protein response (e.g., CALR) were detected in AF, HCAs and HCCs of STAM and STZ control mice. In addition, double immunohistochemistry for CACHD1 and PCNA, p62, or Atg12 in CACHD1^+^ foci and tumors demonstrated, that cell proliferation was elevated but autophagy was suppressed in the CACHD1^+^ area in STAM mice. In response to elevated oxidative stress and DNA damage, mitochondrial prohibitins and YME1L1 overexpression is likely to occur and exert anti-autophagy and anti-apoptotic effects in CACHD1^+^ foci and tumors. Thus, prohibitins act as repressors of transcription via the recruitment of histone deacetylases, and play an important role in the generation of the TGF-β-mediated mesenchymal cell phenotype and suppression of apoptosis [4]. Furthermore, YME1L1, a member of the AAA family of ATPases embedded in the inner mitochondrial membrane, controls the accumulation of respiratory chain subunits in mitochondria and is required for apoptotic resistance, cristae morphogenesis and cell proliferation [32].

Results of proteome analysis of CACHD1 knockdown Huh7 and HepG2 cell lines revealed that *CMYC*, *NMYC* oncogenes and YBX1 and Nrf2 transcriptional factors were inhibited, but TGF-β was activated. From proteome analysis, involvement of CACHD1 in regulation of protein folding, unfolded protein response, autophagy, apoptosis and cytoskeleton organization was predicted. To further investigate the influence of CACHD1 in regulation of the cell cycle, we analyzed mRNA expression of *cyclin D1* and *p21*^WAF1/Cip1^ genes in CACHD1 knockdown human liver cancer cell lines. It was found that the knockdown of CACHD1 in Huh7 and HepG2 cell lines inhibited cell growth and proliferation due to suppression of CD1 and induction of cyclin-dependent kinase inhibitor p21^Waf1/cip1^. From the obtained results, suppression of CD1 and elevation of p21^WAF1/Cip1^ mRNA and cellular proliferation in CACHD1 knockdown human liver cancer cells, could induce p21^WAF1/Cip1^-dependent G1 and G2 arrest, which is related to capacity for direct binding to PCNA and inhibition of cyclin-dependent kinase complexes [33]. In line with our data, decrease of CD1 in Huh7 and HepG2 human liver cancer cell lines by anti-tumor agents was suggested to block CD1 turnover [34].

Assessment of CACHD1^+^ foci number and area STAM mice can help to evaluate the effects of various promoters and inhibitors on NASH-associated hepatocarcinogenesis at different time-points and to investigate early changes and mechanisms in vivo. In our previous study, we assessed CK8/18 in STAM mice [4]. The positive expression was found in the basophilic and eosinophilic foci; however, mixed-cell type foci containing ballooned cells and lipid droplets were CK8/18-negative [4]. In contrast, in this study, CACHD1 was positive in all type of AF, including the mixed-cell type foci and those that were impossible to identify histopathologically. We, therefore, concluded CACHD1 to become the promising marker of NASH-associated preneoplastic lesions in STAM mouse model. In recent studies, this STAM mice NASH model was used to investigate amino acids metabolism, their pharmacological effects and the influence of lipid-lowering agents on NASH and tumor development [35,36]. Furthermore, potent activators of transcriptional regulator Nrf2 with numerous cytoprotective functions were shown to be useful for the treatment of NASH in STAM mice model. For instance, omaveloxolone has been reported to suppress leptin and elevate adiponectin levels in serum and possess antifibrotic activity in the liver [37]. In addition, Liebig et al. discovered that increased n-3 polyunsaturated fatty acids (PUFA) ratios lead to improved survival and attenuated tumor progression in STAM mice, thus, suggesting PUFA to become new therapeutic options against NAFLD-related tumorigenesis [38]. In consequence of these studies, assessment of CACHD1^+^ foci as an early marker of preneoplastic lesions in STAM mice NASH model could become a useful tool to analyze the effects of various potential NASH and tumor inhibitors and promoters in both short- and long-term studies.

The autophagy and ubiquitin-proteasome system are two distinct interacting proteolytic systems that play critical roles in cell survival [21]. We have observed that CACHD1 expression in AF and tumors was coordinated with overexpression of p62 but inversely correlated with the expression of autophagy markers Atg12, Atg7, and activated form of protein kinase R-like endoplasmic reticulum kinase (P-PERK), which is a transmembrane protein kinase of the PEK family [21]. Both p62 and PERK are involved in endoplasmic reticulum stress and regulate autophagy. Thus, p62 is a famous acceptor of autophagy and a multifunctional protein participating in proteasomal degradation of ubiquitinated proteins and different signaling pathways, such as the Keap1-Nrf2 pathway [21]. It was, however, reported that the intracellular level of p62 protein depends on transcriptional regulation and post-translational autophagic degradation [21]. In our study, p62 was overexpressed, but P-PERK and autophagy markers were suppressed in CACHD1^+^ foci, HCAs and HCCs, thus, being indicative of suppressed autophagy. Furthermore, the observation of suppressed P-PERK expression in CACHD1^+^ foci and tumors, but its elevation in the surrounding liver tissue of STAM mice followed reports linking PERK to insulin processing, NASH and its ability to activate autophagy [39]. In previous reports, an increase in unfolded/misfolded proteins in the ER lumen was shown to activate the unfolded proteins response by causing the dissociation of PERK and heat shock protein A5 (HSPA5 (GRP78)), resulting in the activation of transcription factor 6 (ATF6) and inositol-requiring enzyme 1 (IRE1) [40]. After dimerization and autophosphorylation, PERK phosphorylates eIF2α and promotes ATF4 synthesis, which in turn regulates the transcription of Atg12, HSPA5, and the proapoptotic protein DNA-damage-inducible transcript 3 (DDIT3 (CHOP)), thus, activating the autophagy [41].

In conclusion, our results indicate that CACHD1 is an early NASH-associated biomarker of liver preneoplastic lesions and tumors in STAM mice NASH model which could be applied to investigate the mechanisms and potential inhibitors or promoters of DM/NASH-associated hepatocarcinogenesis in this animal model. CACHD1 function is related to control of the cell cycle and autophagy process. The role of CACHD1 in other mice NASH models and human NASH-associated liver cancer is the subject for our further investigations.

## 4. Materials and Methods

### 4.1. Chemicals

Reagents and standards were purchased from Sigma (St. Louis, MO, USA) or Wako Pure Chemicals Industries (Osaka, Japan). All chemicals were of analytical grade.

### 4.2. STAM Mice Experiment

Six-week-old STZ control and NASH-STAM male mice were purchased from Charles River Laboratories, Japan, Inc. (Kanagawa, Japan), where they were generated as described previously [13]. Briefly, on the second day after birth, C57BL/6N mice were subjected to a single subcutaneous (s.c.) injection of 200 μg streptozotocin (STZ) (Sigma, MO, USA), which has been reported to partially damage pancreatic Langerhans islands, cause impaired insulin secretion, and induce insulin resistance and oxidative stress [13,15]. After that, mice were divided into two groups, the STZ (7 mice) group and the NASH-STAM (12 mice) group. Starting from week 4 after the injection and up to the end of the experiment, the NASH-STAM group was administered HFD-60 (Oriental Yeast Co., Tokyo, Japan), while the STZ group was fed with control diet (AIN93G, Oriental Yeast Co., Tokyo, Japan). The administration of HFD to STZ-treated mice induced fat accumulation in the liver, which progressed to NASH in STAM mice. Thereafter, mice were purchased from Charles River Laboratories and housed in plastic cages with wood chips for bedding in animal facility of our university at a constant temperature of 23 ± 1 °C and relative humidity of 44 ± 5% and 12 h (7:00–19:00) light/dark cycle. Animals were given free access to tap water and food. All surviving mice were euthanized under the isofluorane treatment (3 and 5 (10-week-old), and 4 and 7 (18-week-old) mice in STZ and STAM groups, respectively), to allow macroscopic, histopathological, proteome, and immunohistochemical analyses. Mice were fasted overnight before blood collection from the abdominal vein for analysis of serum adiponectin and leptin levels by Elisa (Mouse Adiponectin and Leptin ELISA Kits (ab108785 and ab199082, respectively, Abcam, Tokyo, Japan) was performed. Tumors and surrounding liver sections were prepared and fixed in 10% phosphate-buffered formalin. Using the histopathological analysis in 18-week-old mice, we assessed the NAFLD activity scores (NAS), according to the method of Kleiner et al. [42]. NAS scores were evaluated as a composite parameter based on separate scores for steatosis (0–3), hepatocellular ballooning (0–2), and lobular inflammation (0–3). The Total NAS score is the sum of these separate scores, and values ≥ 5 are correlated with a diagnosis of NASH in humans [41]. Incidences of HCA and HCC, and liver preneoplastic foci (B.F., E.F. and M.F.) were determined. Periodic acid-Schiff stain (PAS) and Azan stain were performed to detect the accumulation of polysaccharides and fibrotic change. Serum fasting blood glucose (FBG) levels were measured weekly by a blood glucose meter (Glutest Ace, Sanwa Chemical, Nagoya, Japan).

### 4.3. Proteome Analysis in STAM Mice HCCs

Quantitative proteome analysis using iTRAQ labeling and QSTAR-Elite LC-Ms/Ms has been performed by the method described previously [4,42]. Shortly, HCCs and liver tissue of 18-week-old STAM and STZ control mice were microdissected from the hematoxylin-stained formalin-fixed and paraffin-embedded (FFPE) liver sections. Due to the difficulties of tumor tissue collection by microdissection and to get the necessary protein concentration (20 μg each), we prepared pooled samples from microdissected HCCs and liver tissue. In our previous study, a comparison of results of LC-Ms/Ms analysis of frozen tissue and FFPE sections showed high concordance [4,43]. Lysis was performed using Liquid tissue lysis buffer (AMR, Tokyo Japan). Duplicate pooled samples from microdissected liver HCCs and livers were labeled as follows: iTRAQ isobaric reagents 114, STAM mouse HCCs; 115, non-tumor liver tissue from STZ control mice. Swiss Protein database (MOUSE) using ProteinPilot™ software (version 2.0, AB Sciex, Concord, ON, Canada) was employed for the analysis of Ms/Ms data with trypsin as the digestion enzyme and methyl methanethiosulfonate for cysteine modification. *P*-value less than 0.05 for protein ratios was considered acceptable. Proteins overexpressed more than 2-fold were considered potential biomarker candidates.

### 4.4. Immunohistochemical Examination

Target proteins were stained in FFPE tissue sections using single and double immunohistochemistry using the standard ABC method by the protocols recently described [33,43]. Briefly, serial formalin-fixed paraffin sections were prepared, and deparaffinization, gradual dehydration and antigen retrieval in citrate buffer (pH 6.0) were performed. Thereafter, sections were incubated with 0.3% (*v*/*v*) hydrogen peroxide for 30 min to inactivate endogenous peroxidase activity. To assess CACHD1 expression in mice liver tissue, the rabbit polyclonal primary antibody (Ab) against CACHD1 (1:500, HPA017202, ATLAS Antibodies, Stockholm, Sweden) was applied overnight at 4 °C. The numbers and areas of CACHD1^+^ foci, and total areas of liver sections, were measured using a color image processor (IPAP; Sumica Technos Osaka, Japan) to give values per cm^2^ of liver section. PCNA mouse monoclonal Ab (1:500, M0879, DAKO, Kyoto, Japan), rabbit monoclonal phospho-mTOR (p-mTOR) (Ser2448) Ab (1:100; Cell Signaling, Danvers, MA, USA), P-PERK (phospho T982) rabbit polyclonal Ab (1:80, ab192591, Abcam, Tokyo, Japan), ATG12 (D88H11) and ATG7 (D12B11) rabbit monoclonal Abs (1:100; ATG12: 4180, ATG7: 8558, Cell Signaling, Danvers, MA, USA), and p62-SQSTM1 rabbit polyclonal Ab (1:300, PM045, MBL Co., Nagoya, Japan), rabbit monoclonal α-SMA Ab (E184) (dilution 1:300; Abcam ab32575, Tokyo, Japan) were employed for the IHC analyses in mice. The 3,3′-diaminobenzidine tetrahydrochloride (DAB) solution (DAKO, Kyoto, Japan) was used for antigen visualization. All immunohistochemical procedures were optimized by testing negative controls and antigen retrieval methods. In double immunohistochemistry, PCNA, TUNEL, p62-SQSTM1, ATG12, P-PERK and P-mTOR were visualized with DAB to get the brown/black color, while CACHD1 was stained blue with alkaline phosphatase (Vectastain ABC-AP kit, Vector blue, Vector Laboratories, Burlingame, CA, USA). Mouse on Mouse Polymer IHC Kit (ab269452, Abcam, Tokyo, Japan) was used for the optimization of background after using mouse monoclonal antibodies. To remove immune complexes after completing the visualization of the first staining with DAB, slides were incubated in 0.2 M glycine (pH 2.2) for 2 h.

### 4.5. In Vitro Experiments

#### 4.5.1. Cell Lines and Culture Conditions

The Huh7 and HepG2 human HCC and COS1 and COS7 cell lines were purchased from the Japanese Collection of Research Bioresources (Osaka, Japan) and routinely maintained in Dulbecco’s modified Eagle’s medium (Invitrogen, Carlsbad, CA, USA) supplemented with 10% fetal bovine serum (FBS; Invitrogen). All cells were incubated at 37 °C in a 5% CO_2_ air-humidified atmosphere.

#### 4.5.2. CACHD1 siRNA Knockdown in Huh7 and HepG2 Human Liver Cancer Cells

CACHD1 expression was transiently knocked down in Huh7 and HepG2 cells using Lipofectamine RNAiMAX (Invitrogen, Carlsbad, CA, USA) according to the manufacturer’s instructions. CACHD1-specific siRNA (Silencer Select siRNA Cat# 4392420; CACHD1 IDs: s33589 (si-CACHD1kn-1), s33590 (si-CACHD1kn-2) and s33591 (si-CACHD1kn-3)) were obtained from Life Technologies (Grand Island, NY, USA). Non-targeting 5 nmol control siRNA (Silencer Select, Cat.No.: 4390843, Ambion, Tokyo, Japan) was obtained from Life Technologies. Huh7 and HepG2 cells (5 × 10^4^/well) were transiently transfected with three types each 6.7 nM CACHD1 siRNAs or control siRNA in a 24-well plate. After 24, 48, 72 and 96 h, si-CACHD1kn-1, and si-CACHD1kn-2 cells were trypsinized and used in Western blot analysis. The best results with a knockdown of CACHD1 were obtained using s33589 (si-CACHD1kn-1) and s33590 (si-CACHD1kn-2); therefore, we used these two siRNAs to perform the Q-RT-PCR, WST-8, and proteome analyses 72 h after transfection.

#### 4.5.3. Real-Time Quantitative PCR

RNeasy Mini Kit (QIAGEN, Carlsbad, CA, USA) was employed for the extraction of total RNA from human liver cancer cells, and the Oligo-dT primer was used for reverse transcription of 1 μg of total RNA. TaqMan probe and primers set for CACHD1 ((Assay ID: Hs 00326087_m1; Public ID: NM_001293274), cyclin D1 (Assay ID: Hs 00277039_m1), p21^Waf1/cip1^ (Assay ID: Hs 00355782_m1), transforming growth factor B2 (TGFB2) (Assay ID: Hs 002342244_m1), and vimentin (VIM) (Assay ID: Hs 00185584) TaqMan Gene Expression Assays (4331182), Thermo Fisher Scientific, Tokyo, Japan)) and the eukaryotic 18S rRNA (4319413E) (Applied Biosystems, Japan) as an internal control, were applied in the real-time RT-PCR analysis of mRNA expressions using ABI Prism 7000 (Applied Biosystems, Foster City, CA, USA) [44]. Data were expressed relative to the number of 18S RNA transcripts. All analyses were performed in triplicate.

#### 4.5.4. Protein Extraction and Western Blot Analysis

The protein extraction from cultivated cells and Western blot analysis were performed as previously described [45] using CelLytic MT (Sigma, St Louis, MO, USA) lysis buffer with protease inhibitor. The total protein amount was detected using the BCA protein assay kit (Pierce), and amounts of 15 μg each were loaded on 10% SDS-polyacrylamide gels and separated. Thereafter, proteins were electrophoretically transferred to polyvinylidene fluoride (PVDF) membranes (Bio-Rad, Inc., Hercules, CA, USA) and probed with primary rabbit polyclonal antibodies against CACHD1 (1:1000, ATLAS Antibodies, Stockholm, Sweden) or β-actin (1:100,000, ab49900, Abcam, Tokyo, Japan) for 1 h at room temperature. After washing, HRP-conjugated secondary antibody (sc-2004, 1:10,000; Santa Cruz Biotechnology, Santa Cruz, CA, USA) was applied. The ECL Plus Western blotting system (GE Healthcare, Piscataway, NY, USA) and LAS1000 image analysis system (Fujifilm, Tokyo, Japan) were used to visualize of immunoreactive bands.

#### 4.5.5. WST-8 Assay

Two CACHD1 siRNAs (6.7 nM) (CACHD1kn-1 and CACHD1kn-2) and control siRNA were transfected into Huh7 and HepG2 human liver cancer cells (1 × 10^4^/well) in a 96-well plate, and the cells were used in assay 72 h later. Filtered solution of water-soluble tetrazolium salt, WST-8 (10 μL), from the Cell Counting Kit-8 (Dojindo Molecular technologies Inc., Kumamoto, Japan) was added to each well and incubated for 1 hr at 37 °C. The converted dye absorbance was detected at 450 nm with background subtraction at 600 nm on microplate reader (Bio-Rad, Tokyo, Japan). All experiments were done in triplicate.

#### 4.5.6. QSTAR LC-Ms/Ms and Ingenuity Pathway Analysis (IPA)

To investigate alterations in protein expression and functional pathways induced by CACHD1 knockdown in vitro, we next performed the proteome analysis in CACHD1kn-2 Huh7 and HepG2 human liver cancer cells (20 μg), and compared with negative controls. DiNa-AI nano LC System (KYA Technologies, Tokyo, Japan) coupled to a QSTAR Elite hybrid mass spectrometer (AB Sciex, Concord, ON, Canada) through a NanoSpray ion source (AB Sciex, Concord, ON, Canada) was employed in the quantitative analysis using iTRAQ reagents, or the non-label analysis as previously described [42]. In vitro knockdown samples were labeled as follows: 114: CACHD1kn-2 Huh7 (HepG2); 115: negative control Huh7 (HepG2) cell lysates. The identification of the proteins from Ms/Ms data was done using the ProteinPilot software 2.0 (AB Sciex, Tokyo, Japan). IPA (Ingenuity Systems, Mountain View, CA, USA) was employed for analysis of protein molecular functions, pathways, and altered up-stream regulators. The transcriptional activation (inhibition) was expressed by the z-score, which value above or lower 2 was considered significant.

### 4.6. Statistical Analyses

All statistical analyses were carried out using StatLight-2000 (C) program (Yukms corp., Kanagawa, Japan). The significance of differences for each parameter was analyzed and evaluated at *p* < 0.05. Statistical analysis with ProteinPilot^TM^ 2.0 Software was employed for the QSTAR Elite LC-Ms/Ms quantitative analysis of protein expression changes in mice HCCs. Data are mean ± SD. The significance of differences between mean values was assessed using the F test. If homogeneous, the data were analyzed with Student’s *t*-test (two-sided), and if not, with the Welch test. Statistical analyses with CACHD1-kn-1 and CACHD1kn-2 Huh7 and HepG2 cells were performed using the Dunnet test.

## 5. Conclusions

In conclusion, CACHD1 is an early NASH-associated biomarker of liver preneoplastic and neoplastic lesions in STAM mice which could be used to investigate the mechanisms and potential inhibitors or promoters of hepatocarcinogenesis in this animal model, and a potential molecular target in DM/NASH-associated liver cancer. CACHD1 expression is likely to be stimulated by hyperglycemia and hyperlipidemia, while its function is related to the regulation of cell proliferation, autophagy and apoptosis in response to oxidative stress.

## Figures and Tables

**Table 1:** Incidence and multiplicity of liver tumors developed in 18-week-old STAM and STZ mice.

Group/Duration	No. Mice	Incidence (%)			Multiplicity (No./Mouse)		
		HCA	HCC	Total	HCA	HCC	Total
STZ	4	0 (0)	0 (0)	0 (0)	0	0	0
STAM	7	7 (100) **	5 (71) *	7 (100) **	2.7 ± 1.7 *	1.0 ± 0.8 *	3.7 ± 2.1 *

* *p* < 0.05; ** *p* < 0.01.

**Table 2:** Top-elevated proteins in HCCs of STAM mice identified by QSTAR Elite LC-Ms/Ms.

Name	GI Number	Ratio	*p* Value	Location	Functions
Cache domain-containing protein 1 (CACHD1)	39930563	3.63	<0.001	C, M	Ca T
Cytokeratin, type II cytoskeletal 8 (CK8)	114145561	2.40	0.0093	C, N	CO, CA, AP
Cytokeratin, type I cytoskeletal 18 (CK18)	254540068	2.33	0.0019	C	CO, CA, AP
Prohibitin 1 (PHB1, PHB)	6679299	2.25	<0.001	Mi, C, N	TR, MiF
Prohibitin 2 (PHB2)	126723336	2.76	<0.001	Mi, C, N	TR, MiF
Glutathione S-transferase Mu 1 (GSTM1)	6754084	3.52	<0.001	C	GM
Peroxisomal bifunctional enzyme (EHHADH)	31541815	2.09	<0.001	P	LM, FABO, PPARS
Thioredoxin-dependent peroxide reductase, mit. (PRDX3)	6680690	2.36	0.013	Mi	ORP, CP, NRA
Ornithine aminotransferase, mitochondrial (OAT)	8393866	2.47	<0.001	Mi	AAM, AC

C, cytoplasm; M, membrane; Mi, mitochondria; N, nucleus; P, peroxisomes. AAM, amino acid metabolism; AC, arginine catabolism; AP, apoptotic process; CA, cell-cell adhesion; Ca T, calcium transport; CO, cytoskeleton organization; CP, cell proliferation; FABO, fatty acid β-oxidation; GM, glutathione metabolism; LM, lipid metabolism; MiF, mitochondrial function; NRA, negative regulation of apoptosis; ORP, oxidation-reduction process; PPARS, peroxisome-proliferating receptor signaling; TR, transcription regulation.

**Table 3:** Incidences of CACHD1-positive and negative preneoplastic and neoplastic lesions in the livers of 18-week-old STAM and control STZ mice.

Group/ Duration	No. Mice	CACHD1^+^ F/BF	CACHD1^+^ F/EF	CACHD1^+^F/MF	Non-BF, EF, MF CACHD1^+^ F	Total CACHD1^+^F/Total AF	Total CACHD1^−^F/Total AF	CACHD1^+^ HCA/ Total HCA	CACHD1^+^ HCC/ Total HCC
STZ/18 w	4	2/3	2/3	7/7	0	12/13	1/13	0/0	0/0
Incidence (%)		66.7	66.7	100	0	92.3	7.7	0	0
STAM/18 w	7	30/33	60/62	107/114	25	222/234	12/234	19/19	7/7
Incidence (%)		90.1	96.8	93.9	-	94.9	5.1	100	100

Data are number of CACHD1^+^ foci, CACHD1^−^ foci, CACHD1^+^ HCA or HCC/number of BF, EF, MF, HCA or HCC, and incidence (%) of CACHD1^+^ lesions. AF, altered foci; CACHD1^+^F, CACHD1-positive foci; CACHD1^−^F, CACHD1-negative foci, BF, basophilic foci; EF, eosinophilic foci; MF, mixed-cell foci; Non-BF, EF, MF, altered foci non-detectable as basophilic, eosinophilic and mixed-cell type by H&E staining; HCA, hepatocellular adenoma; HCC, hepatocellular carcinoma.

**Table 4:** Proteins with significantly inhibited expression in CACHD1 kn Huh7 and HepG2 liver cancer cells.

Name	ID	CACHD1kn Huh7/HepG2	Location	Type	Function
Protein folding and unfolded protein response					
Cache domain-containing 1 (CACHD1)	14285643	↓/↓	U	O	CH
Calreticulin (CALR)	117501	↓/↓	C, EPR	TR	PF, UPR, TR
Calumenin (CALU)	5921197	↓/↓	C, EPR	O	PF, UPR,CPMP
Heat shock 70kDa protein 2 (HSPA2)	1708307	↓/↓	C, EPR	O	UPR
Heat shock 70kDa protein 9 (mortalin) (HSPA9)	21264428	↓/↓	C, EPR	O	UPR
Heat shock 70kDa protein 5 (Glu-regul. protein 78kDa) (HSPA5)	14916999	↓/↓	C, EPR	E	UPR
Cytoskeleton organization					
Cytokeratin 8 (CK8)	90110027	↓/↓	C	O	CO
Cytokeratin 18 (CK18)	125083	↓/↓	C	O	CO
Cytokeratin 19 (CK19)	311033484	↓/↓	C	O	CO
Actin beta like 2 (ACTBL2)	172046825	−2.13/−2.05	N	O	ACO
ENAH actin regulator (ENAH)	48428086	−2.38/−2.13	PM	O	ACO
Myristoylated alanine-rich protein kinase C substrate (MARCKS)	76803798	↓/↓	PM	O	ACO
Cofilin 1 (non-muscle) (CFL1)	116848	↓/↓	N	O	ACO
MARCKS-like 1 (MARCKSL1)	1346576	↓/↓	C	O	ACO, Ca T, CP
Profilin 1 (PFN1)	130979	↓/↓	C	O	ACO
Tubulin, alpha 1c (TUBA1C)	20455322	↓/↓	C	O	CO, CD, CDIT
Stress response, apoptosis, autophagy					
Superoxide dismutase 2, mit. (SOD)	134665	↓/↓	C	E	NRAP, MiF
Y box binding protein 1 (YBX1)	54040030	↓/↓	N	TR	NRAP; NRCS
Nucleophosmin (numatrin) (NPM1)	114762	−2.2/−2.0	N	TR	NRAP, CP; CCP
Alpha-fetoprotein (AFP)	120042	−3.02/−2.75	ES	T	CPM
Nucleolin (NCL)	90110781	↑/↑	N	O	A
Lemur tyrosine kinase 3 (LMTK3)	117949603	↑/↑	O	K	A, AP
Prolyl endopeptidase (PREP)	215273868	↓/↓	C	P	A
Epoxide hydrolase 1, microsomal (xenobiotic) (EPHX1)	123926	−2.30/−2.12	C	P	XM
Aspartate aminotransferase 2 (GOT2)	308153643	↓/↓	C	E	AAM, FAT

Data are mean fold-changes of all differentially expressed proteins in subjected to analysis Huh7/HepG2 CACHD1 knockdown cell lines. ↓,↑: down-regulated or up-regulated, respectively, in non-label LC-Ms/Ms analysis (no expression was detected in negative control cell lines). A, autophagy; AP, apoptosis promotion, AAM, aminoacid metabolism; ACO, actin cytoskeleton organization; Ca T, calcium ion transport; CO, cytoskeleton organization; CD, cell division; CH, calcium homeostasis; CP, cellular proliferation; CCP, cell cycle progression; CDIT, cytoskeleton-dependent intracellular transport; CPM, cellular protein metabolism; FAT, fatty acid transport; MiF, mitochondrial function; NRAP, negative regulation of apoptotic process; NRCS, negative regulation of cellular senescence; UPR, unfolded protein response; XM, xenobiotic metabolism. C: Cytoplasm; CS: Cytoskeleton; ES: Extracellular space; N: Nucleus; PM: Plasma membrane; U: unknown; TR: transcriptional regulator; E: Enzyme; K: Kinase; P: Peptidase; T: transporter; O: other.

## Data Availability

Data is contained within the article or supplementary material.

## Supplementary Materials

The following are available online at https://www.mdpi.com/2072-6694/13/6/1216/s1, Figure S1: Reduction of CACHD1 protein level in both Huh7and HepG2 cells with the transfection of si-CACHD1kn-1 and si-CACHD1kn-2.
