# Supplementary material for: Cache Domain Containing 1 Is a Novel Marker of Non-Alcoholic Steatohepatitis-Associated Hepatocarcinogenesis
Source: Cancers (Basel). 2021 Mar 10;13(6):1216. doi: 10.3390/cancers13061216 (PMC8001421; doi:10.3390/cancers13061216)
Supplement: Supplementary file 1 [file cancers-13-01216-s001.pdf]

# Supplementary Material: Cache Domain Containing 1 is a Novel Marker of Non-Alcoholic Steatohepatitis-Associated Hepatocarcinogenesis

**Anna Kakehashi, Arpamas Chariyakornkul, Shugo Suzuki, Napaporn Khuanphram, Kumiko Tatsumi, Shotaro Yamano, Masaki Fujioka, Min Gi, Rawiwan Wongpoomchai and Hideki Wanibuchi**

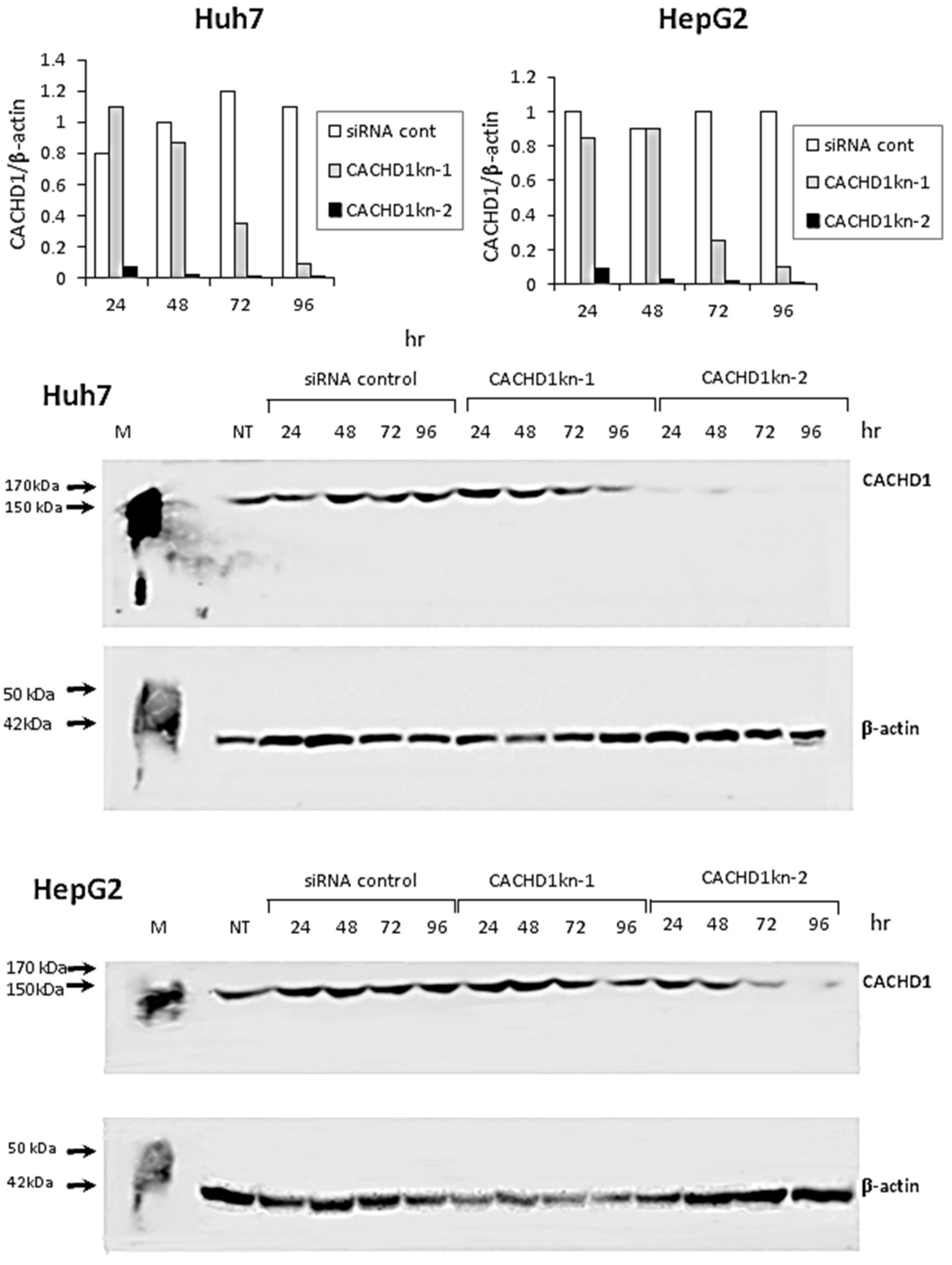


NT, non-treated

**Figure S1.** Reduction of CACHD1 protein level in both Huh7and HepG2 cells with the transfection of si-CACHD1kn-1 and si-CACHD1kn-2. Figure also contains the uncropped Western Blots of Figure 3A.

**Publisher's Note:** MDPI stays neutral with regard to jurisdictional claims in published maps and institutional affiliations.

© 2021 by the authors. Licensee MDPI, Basel, Switzerland. This article is an open access article distributed under the terms and conditions of the Creative Commons Attribution (CC BY) license (http://creativecommons.org/licenses/by/4.0/).
